# Assessment and Validation of Small-Scale Tropospheric Delay Estimations Based on NWP Data

**DOI:** 10.3390/s24206579

**Published:** 2024-10-12

**Authors:** Jan Erik Håkegård, Mohammed Ouassou, Nadezda Sokolova, Aiden Morrison

**Affiliations:** 1 SINTEF Digital, Strindveien 4, 7032 Trondheim, Norway; nadia.sokolova@sintef.no (N.S.); aiden.morrison@sintef.no (A.M.); 2 Norwegian Mapping Authorities, Kartverksveien 21, 3507 Hønefoss, Norway; ouabobaka@gmail.com

**Keywords:** GNSS, NWP, tropospheric gradient

## Abstract

This paper investigates the applicability of the Numerical Weather Prediction (NWP) data for characterizing the gradient of zenith wet delay in horizontal direction observed on short baselines over larger territories. A three-year period of data for an area covering Scandinavia and Finland is analyzed, and maximum gradients during the considered period are identified. To assess the quality of the NWP-based estimates, results for a smaller region are compared with the estimates obtained using Global Navigation Satellite System (GNSS) measurements processed by the GipsyX/RTGx software package (version 2.1) from a cluster of GNSS reference stations. Additionally, the NWP data from 7 to 9 August 2023 covering a period that includes a storm with high rain intensities over Southern Norway leading to sustained flooding are processed and analyzed to assess if the gradient of zenith wet delay in the horizontal direction increases significantly during such events. The results show that maximum gradients in the range of 40–50 mm/km are detected. When comparing NWP-based estimates to GNSS-based estimates, the tropospheric delays show a very strong correlation. The tropospheric gradients, however, show a weak correlation, probably due to the uncertainty in the NWP data exceeding the gradient values. The data captured during the storm show that while the tropospheric delay increases significantly it is difficult to see increases in the gradient of zenith wet delay in the horizontal direction using this data source and resolution.

## 1. Introduction

The accuracy of Global Navigation Satellite System (GNSS)-based positioning can be impacted by atmospheric effects, i.e., tropospheric and ionospheric delays. The troposphere, the lowest layer of the Earth’s atmosphere is characterized by significant spatial and temporal changes in temperature, pressure, and water vapor content, all of which influence the propagation of radio signals from GNSS satellites. As discussed in [1], these variations can be linked to water vapor distribution and evolution of the precipitating system dynamics and behavior. In general, the tropospheric delay phenomenon is well understood. In nominal conditions, the majority of this delay can be calculated and eliminated by applying a tropospheric model as detailed in [2,3], through using differential processing implementations such as the Real Time Kinematic (RTK), by applying State Space Representations (SSR) corrections [4,5] developed in support of the high precision techniques such as the Precise Point Positioning PPP-RTK [6], or through other approaches. However, weather events such as thunderstorms, intense precipitation and local convective clouds can lead to increased spatial and temporal variation in the tropospheric delay [7,8]. In multiple GNSS-based positioning techniques, the tropospheric corrections are generated based on values obtained from a number of reference stations/receivers and interpolated to the approximate user position. While numerous interpolation techniques have been proposed [9], accurate modeling of the tropospheric delay’s spatial variation during anomalous tropospheric events remains a challenge. As indicated by multiple authors [10,11], the unmodeled residuals degrade the solution accuracy by impacting the carrier-phase ambiguity resolution (AR) process. Additionally, if integrity is supported by the system, such errors if passed unnoticed might pose an integrity threat. The importance of characterizing and quantifying the variations as well as approaches to account for the impact of anomalous tropospheric disturbances have been addressed in several previous works [10,12,13,14,15].

There is also an existing body of research focusing on the spatiotemporal variability characterization of the lower troposphere on both global and regional scales through using coarse spatial grids [14,16,17]. In several systems/applications, the critical element with the most stringent accuracy and integrity requirements (if supported) is a short distance (5–10 km) separating the user from the ground system/virtual reference station location, etc. The development of high-density numerical weather models (NWMs) has initiated multiple studies on how these models can be used to generate tropospheric products that can assist positioning techniques and services [18,19,20]. The potential use of numerical weather prediction (NWPs) for the development of parametric threat models for tropospheric anomalies in support of high integrity systems has also been considered due to the possibility of performing characterization of tropospheric spatial variation with very high resolution over larger territories [21,22].

This study pursues the horizontal tropospheric gradient analysis over the Scandinavian countries, Finland and the surrounding areas. It uses NWP data from the Norwegian Meteorological Institute (MET Norway) over a three-year period with a spatial resolution of 2.5×2.5 km as a basis to support applications that rely on short baseline performance. As the high-resolution NWP-data-based results might suffer from the residuals due to features not being captured by the model, to assess the achieved accuracy the obtained values are also compared with GNSS-derived measurements over a dense cluster of monitoring receivers located in the north of Norway.

In August of 2023, the southern regions of Norway, parts of Sweden, Finland and the Baltics experienced a sequence of severe weather events over several days collectively referred to as ’Hans’. To investigate the levels of spatial and temporal variation in the tropospheric delay during such intense and dynamic tropospheric conditions as are contained in such a storm event, this paper undertakes analyzing the NWP data for the region of Norway affected most.

## 2. Finding Maximum Tropospheric Gradients within the Area of Interest

### 2.1. Description of the Data

The NWP data used in this study are collected from MET Norway’s Thematic Real-time Environmental Distributed Data Services (THREDDS) server https://thredds.met.no/ (accessed on 7 August 2024). One of the products that are openly available is the MetCoOp Ensemble Prediction System (MEPS) covering Scandinavia and Finland. MEPS provides 30 ensemble members every six hours as well as a MEPS Deterministic that refers to a single specific forecast produced by one of the simulations within the MEPS. It is the most likely outcome predicted by that particular simulation, and the data used in this work.

**Figure 1:**
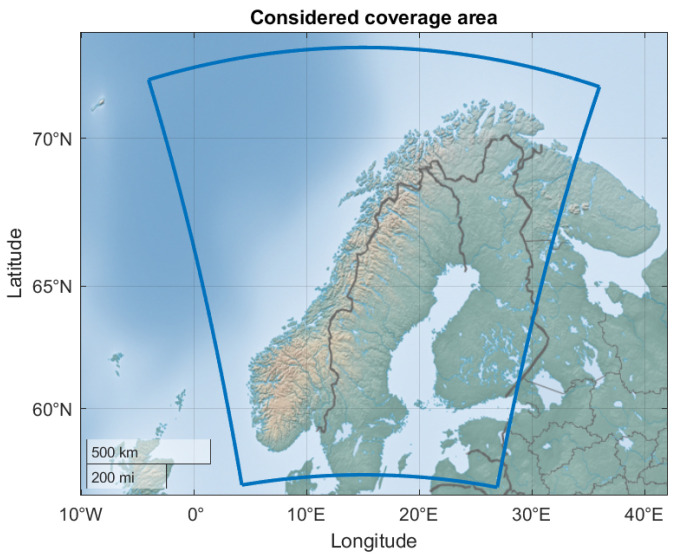
Area considered including Norway, Sweden and Finland marked by the blue line.

The coverage area included in this study contains a part of the MEPS coverage and is shown as the blue bounding box in Figure 1. The spatial resolution of the NWP data is 2.5×2.5 km and the size of the grid is 560×700. The MEPS data contain 3D meteorological parameters for a number of vertical model levels. The model level height is a hybrid that follows the ground near the surface, and gradually converts to pressure level coordinates higher in the troposphere. It depends on the model level temperature and pressure.

### 2.2. Methodology of Estimating the Tropospheric Gradient

In order to estimate the tropospheric gradient based on NWP data, we first calculate the zenith tropospheric delay (ZTD) at each of the grid points. The procedure employed to conduct this is described in [21]. The ZTD is composed of two components; the zenith wet delay (ZWD), and the zenith hydrostatic delay (ZHD). The ZHD can be accurately estimated using well-known models. The ZWD may be more difficult to estimate, in particular during severe weather conditions. In the following, we are, therefore, concentrating on the ZWD.

In this study, we are concerned with the horizontal gradients. Therefore, the ZWD is estimated for several heights above sea level in addition to at the surface. The reason for this is that in mountainous areas with abrupt changes in terrain height, only using surface data may lead to gradients due to the difference in height and not due to the troposphere.

The first-order approximation of the gradient between two pixels is calculated as the difference in ZWD (dZWD) divided by the distance between the two stations.

**Figure 2:**
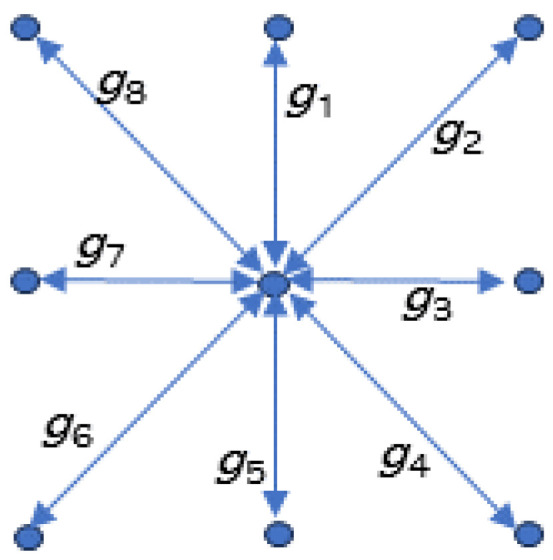
Illustration of the eight gradients calculated for each pixel.

The maximum gradient for each pixel (except for the edge pixels) is estimated by finding the maximum gradient among the eight gradients that can be calculated towards the neighboring pixels (see Figure 2). Hence, the derived maximum gradient at time *t* for each pixel (m,n) is as follows:

(1) $$g_{max}\left(m,n,t\right)=\max_{i}\left(g_{i}\left(m,n,t\right)\right),m=2,\ldots ,559,n=2,\ldots ,699,i=1,\ldots 8$$

It is easy to see that, considering the entire coverage area, the gradients towards pixels further away can never be larger than the gradients towards neighboring pixels. Hence, in this way, we find the maximum gradients within the coverage area according to the NWP data.

The maximum gradient for the entire coverage area for each time sample is found:

(2) $$g_{MAX}\left(t\right)=\max_{m,n}(g_{max}\left(m,n,t)\right)$$

### 2.3. Results

**Figure 3:**
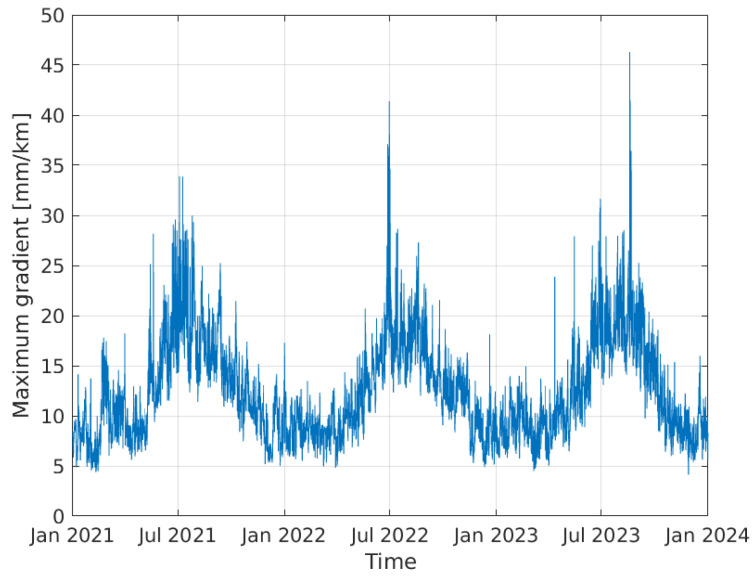
Timeseries of the maximum estimated tropospheric horizontal gradient versus time for three years of data.

**Figure 4:**
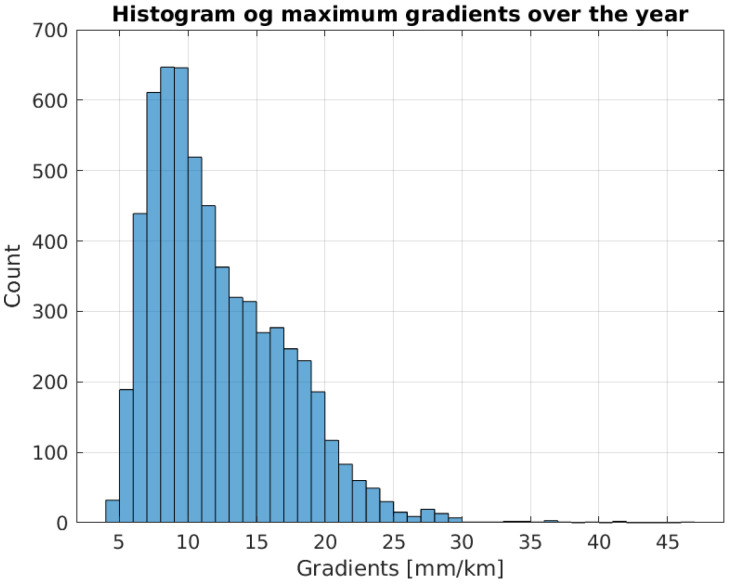
Histogram of the maximum estimated tropospheric horizontal gradient for the three years of data.

The resulting timeseries for the maximum gradient for the years 2021–2023 is shown in Figure 3. The histogram of the values is shown in Figure 4. Many of the values are below 10 mm/km, while the maximum value is above 40 mm/km. As expected, the maximum gradients occur during the summer months of June, July and August.

**Figure 5:**
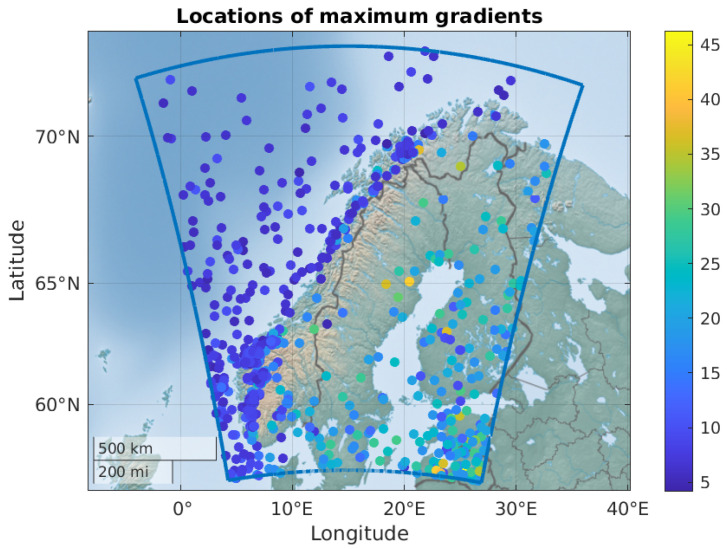
Illustration of where the maximum tropospheric horizontal gradients are found. The color of the dots indicates the size of the gradient in mm/km.

Figure 5 shows where the maximum gradients are found. The density of dots is highest in the southwestern part of the coverage area, and generally higher over sea and close to the sea than over land far from the sea. This is not very surprising, as the humidity typically is higher over the sea, leading to more rapid variations in tropospheric delay.

Figure 5 also illustrates the magnitude of the maximum gradients. We see that the maximum gradients that are located at sea generally are low, and the steepest gradients occur over land. Steep gradients occur when the specific humidity in the lowest 10 km of the atmosphere varies over short distances. The other two parameters of importance (pressure and temperature) do not vary over short distances to the same extent, as shown in [21].

## 3. Validation of the Tropospheric Gradient

### 3.1. Data Processing

To assess the performance of the proposed algorithms, we employed the GipsyX/RTGx software version 2.1 package developed by the Jet Propulsion Laboratory (JPL). This next-generation software is designed for applications in positioning, navigation, timing, and Earth science. It utilizes measurements from three geodetic techniques: Global Navigation Satellite Systems (GNSS), Satellite Laser Ranging (SLR), and Doppler Orbitography and Radiopositioning Integrated by Satellite (DORIS). The software has been completely redesigned to improve accuracy, precision, and flexibility. For further details, readers can refer to [23].

The package estimates time-dependent parameters (TDP), including satellite clock biases, tropospheric wet zenith delay, horizontal tropospheric gradients in the east and north directions, station position, and velocity. The algorithm used by GipsyX to compute the gradient is detailed in [24].

We processed all the stations within the area of interest, obtaining values for the tropospheric wet zenith delay and the horizontal gradient with a sampling rate of five-minute intervals. We then applied the resampling/bootstrapping algorithm [25,26] to enhance the performance of the validation process.

We configured the software to compute the troposphere values as follows. The tropospheric model used is the Global Mapping Function (GMF). The apriori value for the troposphere wet delay is set to 0.1 m by default and is modeled as a random walk [27] (p. 127). For the gradients in the east and north directions, the apriori values and the starting sigma values are also modeled as random walks.

The computation of the troposphere gradient of the wet zenith delay proceeds as follows. Let $V_{i}$ and $V_{j}$ represent the troposphere wet zenith delays observed at two arbitrary reference stations, and let $t_{k}$ be the epoch at which the gradient $G_{i,j,k}$ is computed. The gradient is calculated using the following equation:

(3) $$\begin{array}{c} G_{i,j,k}=\frac{V_{i}-V_{j}}{\sqrt{{\left(x_{1}-x_{2}\right)}^{2}+{\left(y_{1}-y_{2}\right)}^{2}}} \end{array}$$

where *x* and *y* are the station coordinates.

### 3.2. Bootstrapping

Because our dataset contains only twelve observations per hour, we use bootstrapping to enhance the reliability of our statistical estimates. With such a small dataset, traditional estimation methods can yield unreliable results. Bootstrapping improves the robustness of these estimates by approximating the distribution of a statistic (such as the mean or variance) through resampling.

The observed data points, denoted as $X=\{x_{1},x_{2},\ldots ,x_{n}\}$, are used to compute the statistic of interest, S(X). A bootstrap sample, $X^{*}=\left\{x_{1}^{*},x_{2}^{*},\ldots ,x_{n}^{*}\right\}$, is generated by randomly selecting *n* data points with replacements from the original dataset X.

The bootstrap algorithm proceeds by generating *B* independent bootstrap samples $\left\{X^{*1},X^{*2},\ldots ,X^{*B}\right\}$, each of size *n*.

Let $S(x^{*b})$ represent the statistic of interest for the *b*-th bootstrap sample. In our case, we are interested in computing the mean and evaluating the quality of our estimation. The sample mean without bootstrapping is given by the following:

(4) $$\hat{\mu }=\frac{1}{n}\sum_{i=1}^{n}x_{i}$$

The bootstrap mean is calculated as:

(5) $${\hat{\mu }}_{B}=\frac{1}{B}\sum_{b=1}^{B}{\hat{\mu }}_{b}$$

where ${\hat{\mu }}_{b}=\frac{1}{n}\sum_{i=1}^{n}X_{i}^{*}$ is the mean of the *b*-th bootstrap sample.

We use bootstrapping to improve the estimation of $V_{i}$ and $V_{j}$, as defined in Equation (3). The quality of our estimation is assessed by computing the bias, standard error, and confidence interval. The bias is estimated as:

(6) $$\mathrm{Bias}={\hat{\mu }}_{B}-\mu$$

where ${\hat{\mu }}_{B}$ is the mean of the bootstrapped statistics and μ is the true value of the parameter.

The standard error of the bootstrap mean is calculated as the standard deviation of the bootstrap means:

(7) $$\mathrm{SE}=\mathrm{Std}({\hat{\mu }}_{b})$$

A confidence interval for the mean is given by the following:

(8) $$\mathrm{CI}={\hat{\mu }}_{B}\pm z\cdot \mathrm{SE}$$

where ${\hat{\mu }}_{B}$ is the bootstrap mean, *z* is the z-score corresponding to the desired confidence level, and SE is the standard error of the bootstrap mean. In this analysis, we used a 95% confidence level.

### 3.3. Heywood Effects

**Figure 6:**
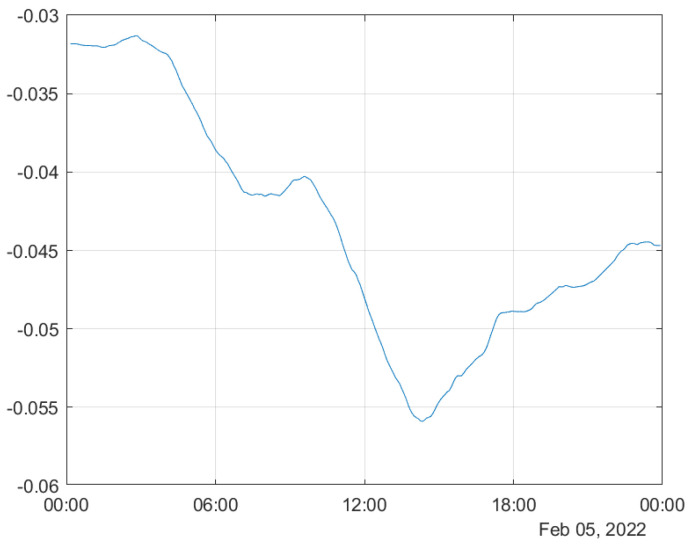
Negative troposphere wet zenith delay.

During our analysis, negative values were observed in the Troposphere Wet Zenith Delay (TWZD) from day of year (DOY) 37 to 57 in 2022 (see Figure 6). These negative values are the result of filter instability, commonly referred to as the Heywood effect, where variances (diagonal elements of the error covariance matrix) become negative during the iterative estimation and updating process. This instability is particularly associated with the Kalman filter, which is employed in the GipsyX software package. Although the Kalman filter is widely used due to its efficiency in handling time-dependent parameters, it can be sensitive to issues such as improper initialization, misconfiguration, or inaccuracies in process noise modeling. These issues can lead to filter divergence, contributing to instability and resulting in negative variance estimates, as observed in the Heywood effect.

This phenomenon is often caused by uncertainties in the estimation process, where small errors in input data or misconfigured settings can lead to significant instabilities. Additionally, improper initialization of key variables or inaccuracies in process noise modeling can exacerbate the problem, resulting in negative variances. The combination of apriori wet delay values, calculated from empirical models, and residual delays estimated during parameter fitting can also produce both positive and negative estimates. However, both dry and wet delays are physically constrained to be positive, with total delays typically amounting to approximately 2.3 m—90% due to the dry component and 10% to the wet component.

To address these issues, researchers have proposed various techniques, such as stabilized estimation filters (e.g., Joseph’s filter), which prevent the occurrence of negative variances by adding constraints to the estimation process.

The occurrence of Heywood effects is not unique to navigation systems. Similar issues are frequently encountered in fields like the social sciences, particularly in methods like factor analysis (FA) and structural equation modeling (SEM), where improper solutions arise when variance estimates exceed one or fall below zero due to high correlations. Ongoing research has identified several contributing factors, including outliers [28], non-convergence, under-identification [29], empirical under-identification [30], and structurally misspecified models [31].

In navigation and surveillance applications, similar challenges arise when using estimation filters for time-dependent parameters. These problems can often be traced back to uncertainties in process noise, ill-conditioned transition matrices (where minor changes can cause significant effects), and improper initialization of apriori statistics. To mitigate these effects, experienced modelers recommend several techniques, such as state-noise compensation, Joseph’s stabilized estimation filters, square-root filtering methods like the Potter–Schmidt filter, and fading memory filters [32,33,34,35].

### 3.4. Cluster of Reference Stations

**Figure 7:**
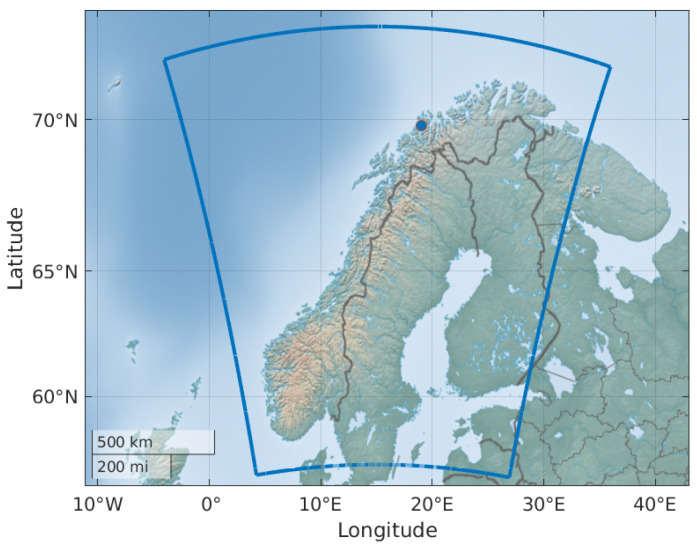
Locations of reference stations in the Tromsø cluster marked with a blue dot within the coverage area of the NWP data marked by the blue line.

**Figure 8:**
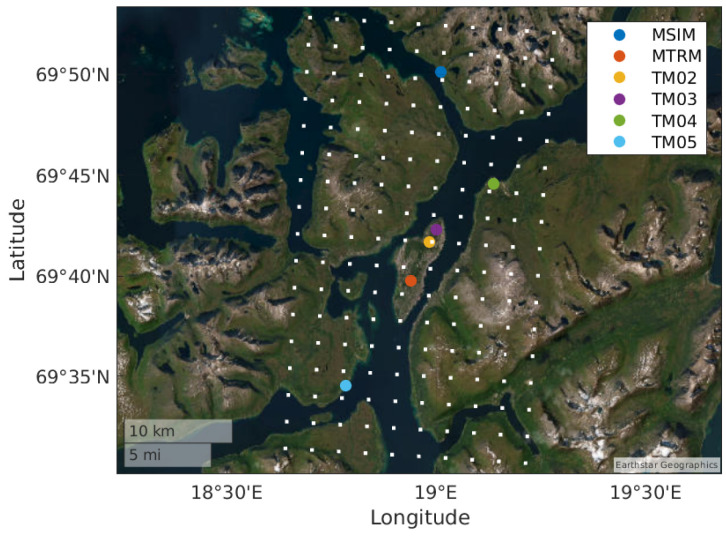
Locations of reference stations on map. Surrounding grid points of the NWP data are also included.

The cluster of reference stations is located in Northern Norway close to Tromsø, as illustrated by the blue dot in Figure 7. The locations of the reference stations are listed in Table 1 and they are shown on a smaller scale map in Figure 8. This figure also contains the surrounding grid points of the NWP data. We see that TM02 and TM03 are so close to each other that they have the same closest grid point. In this section, only data from 2022 are used.

The baseline distances between the stations are listed in Table 2. They range from 1.3 km to 30.3 km. Table 3 contains the distances between the respective closest grid points. Table 4 contains the distances from each reference station to the closest grid point. We see that the distances vary from 285 m to 1437 m. (The maximum possible distance to the closest grid point is 1768 m). The table also contains the surface height of the closest grid point to compare it to the height of the reference stations. We see that the heights of, in particular, MTRM, TM02 and TM03 are quite different from the surface height of the closest grid point.

When evaluating and comparing gradient estimates based on NWP data and GNSS data, the vertical and horizontal differences in location must be considered:

- We have seen that the height of the reference stations sometimes is different from the surface height of the closest grid points. It is, therefore, important to calculate the ZWD based on NWP data using the height of the reference station, and not the surface height of the grid points. As the gradients typically are in the sub-mm/km area, not doing this may introduce significant errors.
- There are no NWP grid points exactly at the locations of the reference stations. The gradients estimated based on GNSS and NWP data will, therefore, be calculated with different baselines, introducing an error in the comparison. The error will increase with the distance between the reference station and the closest grid point. To reduce this error, spatial interpolation is applied to align the location of the NWP-based estimates and the GNSS-based estimates.

### 3.5. Comparing ZWD Estimates

Before comparing gradients estimated from GNSS data and from NWP data, we first compare ZWD estimates. If the ZWD data are uncorrelated, there will not be any correlation between gradients either.

The temporal resolution of the GNSS data is 5 min. The reference time is 1 January 2000 11:59:47. The timestamps in the files are the number of seconds after the reference time. The temporal resolution of the NWP data is as mentioned earlier 6 h at the beginning of the year, and then 3 h for the rest of the year. In order to compare the two timeseries, the GNSS-based data are, therefore, decimated and resampled onto the NWP timestamps.

**Figure 9:**
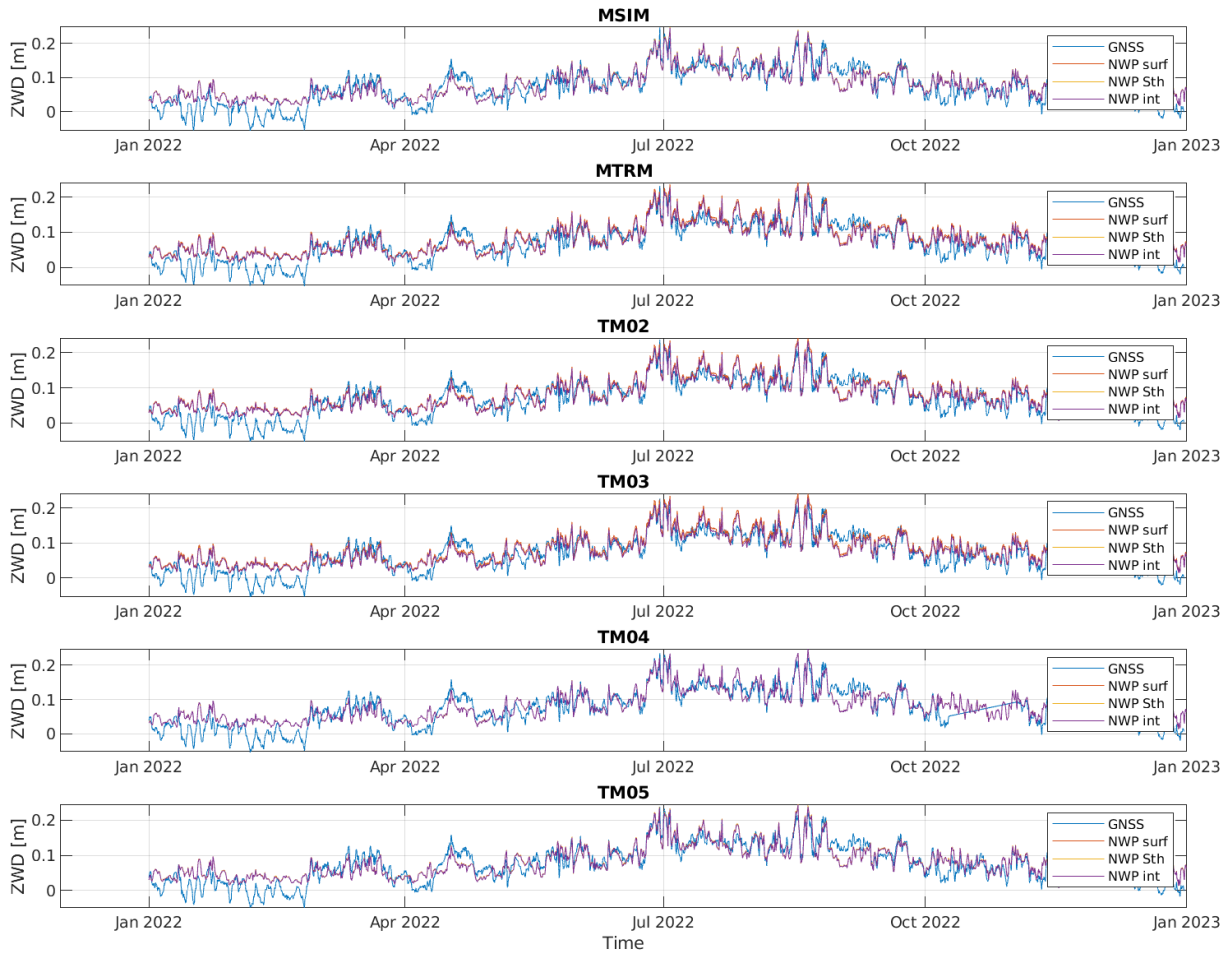
Timeseries of ZWD values from GNSS measurements and NWP data at the six reference stations from 1 January 2022 to 1 January 2023.

Figure 9 shows timeseries of ZWD data based on GNSS and NWP data. As mentioned in Section 3.3, some of the GNSS-based ZWD values are negative. This is physically impossible, but common as a modeling artifact with similar results reported in other publications. In [36], negative ZWD estimates from the GipsyX software are detected. The cause is assumed to be ZHD modeling errors, as the ZWD is calculated as the difference between the estimated ZTD and the estimated ZHD. The same explanation is given in [37], while in [38] it is considered that it might also be related to inappropriate ZTD estimation methods. In any case, these physically impossible results are obviously artifacts of modeling, and while they could be eliminated by re-basing the minimum observed result to zero, this step is seen as unnecessary for further analysis.

Three different curves for NWP-based ZWD data are included, to see the effect of using reference station height instead of surface height and using horizontal interpolation. These are as follows:

- NWP surf: The ZWD is estimated at the closest grid point and at surface height.
- NWP Sth: The ZWD is estimated at the closest grid point but at the height of the reference station.
- NWP Int: The ZWD is estimated at the reference station coordinates using 2D interpolation and the height of the reference station.

**Figure 10:**
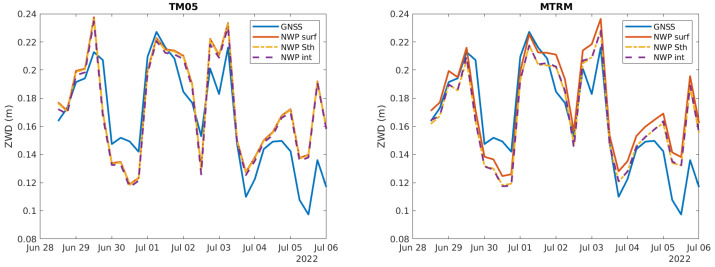
Zoomed in version of ZWD curves for two stations.

As it may be difficult to see the difference between the curves in Figure 9, a zoomed-in version of the curves for two of the stations is shown in Figure 10. For MTRM, the station height is about 134 m above the surface height, so the estimated ZWD based on surface height is higher than the ZWD based on the reference station height. For TM05, the station height is about the same as the surface height, but the distance to the closest grid point is over 1.2 km. Therefore, the ZWD using interpolation is slightly different from the other two curves based on NWP data. The blue curve corresponding to the ZWD estimated based on GNSS data is correlated with the other three curves, although there is not a perfect match.

The mean values of the ZWD for the timeseries are shown in Table 5. We see that the estimates using NWP data are some mm higher than the estimates based on GNSS data, which might suggest the GNSS-based data have a systemic negative bias. The mean value using surface data is some mm higher than the mean value using station height for those stations with a difference between station and surface height as expected. There are minor differences in the mean value when applying interpolation. This is also as expected, as the variation in ZWD between neighboring grid points is small and the error we have by not using interpolation will probably have close to zero mean.

The mean and standard deviation of the difference between ZWD estimates based on GNSS and NWP data are shown in Table 6 and Table 7, respectively. The difference in mean value is largest using surface height, and lowest using interpolated data. The standard deviation is pretty much the same for the three methods.

**Figure 11:**
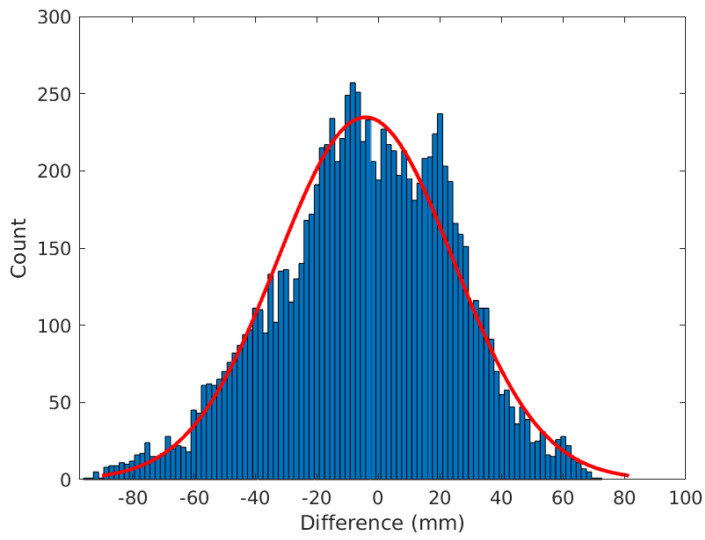
Histogram over the difference between the ZWD estimated using GNSS and using NWP with interpolation for all stations together. The red curve indicates the best fit to a normal density function.

Figure 11 shows the histogram for the difference between ZWD estimates based on GNSS data and estimates based on NWP data and interpolation including all six stations. For most of the data samples, the difference is within 50 mm, while for some it is as large as 100 mm.

Of the two methods to estimate the ZWD, it is expected that the GNSS-based method is the most accurate one. The NWP-based ZWD estimates are based on the humidity, pressure and temperature along a vertical column of the troposphere. These parameters are estimated and not measured. The estimation is also conducted ahead of time and is, therefore, a prediction. The humidity field is highly variable, in particular, in severe weather conditions, introducing an error in the estimates of the ZWD. It is expected that the GipsyX software contains algorithms that reduce the error in the GNSS-based estimates to a level significantly below the error of the NWP-based estimates.

As a conclusion of this section, the ZWD estimates based on NWP data show a good correlation with ZWD estimates based on GNSS data. It might, however, be that the ZWD estimates based on GNSS have a small but measurable systemic negative bias. When estimating ZWD using NWP data, it makes a significant difference to use the reference station height and not the surface height. Some further improvements may be obtained using interpolation.

#### Correlation Coefficient of ZWD Data

Another way of assessing how different timeseries are is to use the Pearson correlation coefficient:

(9) $$\rho_{\left(X,Y\right)}=\frac{cov(X,Y)}{\sigma_{X}\sigma_{Y}}$$

How to interpret the correlation coefficient is shown in Table 8.

Table 9 shows how correlated the NWP-based ZWD estimates at the different reference stations are. They are highly correlated, and most correlated at reference stations that are closely located such as MTRM, TM02 and TM03. Table 10 shows that the ZWD estimates based on GNSS also are correlated at the different reference stations, similar to the NWP-based estimates.

Table 11 shows the correlation between the NWP-based and GNSS-based ZWD estimates. It is about 0.82 for all stations. Hence, there is a very strong correlation, but still much less than the correlation between the ZWD estimates at the different reference stations.

If we assume that the GNSS-based estimates are accurate, the curves and tables in this section indicate that using NWP data to estimate the ZWD has a relatively good accuracy. If it is good enough will depend on the application. The next step will be to see if NWP data also can be used to estimate gradients with good accuracy.

### 3.6. Comparing Gradient Estimates between Stations

The first-order approximation of the gradient between two stations is calculated in a similar way as in the previous section (see Equation (3)). With six stations, a total of 15 gradients are calculated in this way.

#### 3.6.1. Timeseries, First Order Statistics of Gradients and the Dependence of Baseline

**Figure 12:**
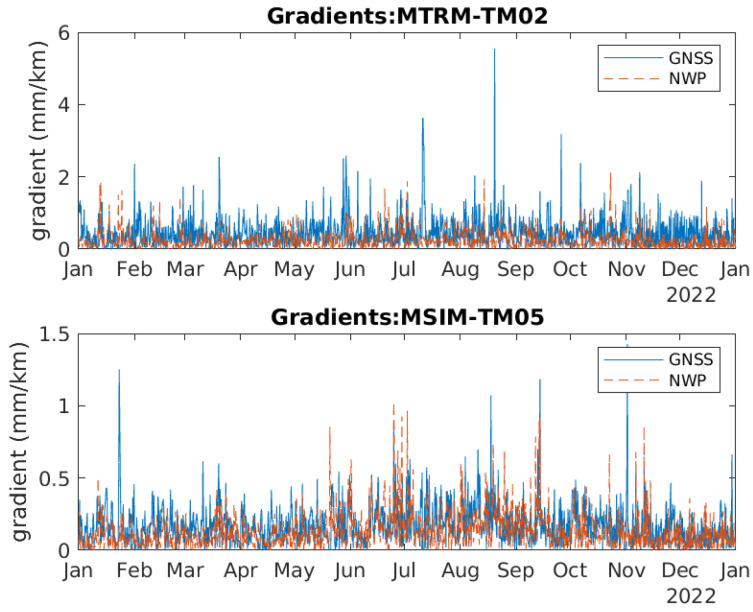
Timeseries of two tropospheric horizontal gradients between two pairs of reference stations (Top: MTRM-TM02. Bottom: MSIM-TM05).

**Figure 13:**
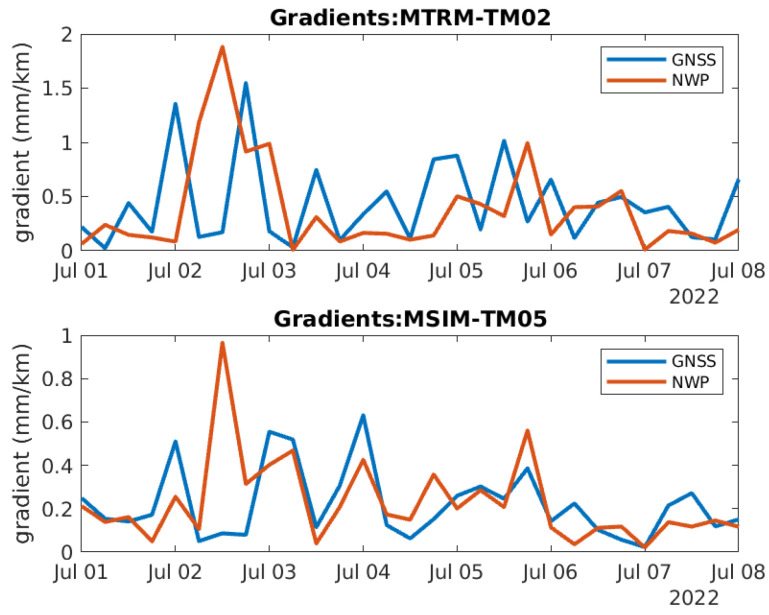
Zoomed in version of Figure 12.

Figure 12 shows the timeseries of the gradients between MTRM and TM02 and between MSIM and TM05. These two gradients are selected as the baseline of the first one is 3.9 km and the baseline of the last one is 30.3 km. They may, therefore, be used to assess the effect of baseline length. The blue curves correspond to the gradients calculated using GNSS data and the red curves to the gradients calculated using NWP data. Figure 13 contains zoomed-in versions of the curves in Figure 12 to better visualize the difference between the curves. Based on the figures, the correlation between the blue and red curves is uncertain, so this is looked further into.

The mean value of the gradients based on GNSS data and NWP data are included in Table 12 and Table 13, respectively. The numbers seem quite correlated. One general observation is that gradients for long baselines, such as MSIM-TM05 and TM04-TM05, have small mean values and gradients for short baselines like TM02-TM03 have large mean values. Another observation is that gradients between stations at different heights, such as TM03-TM04, have large mean values.

**Figure 14:**
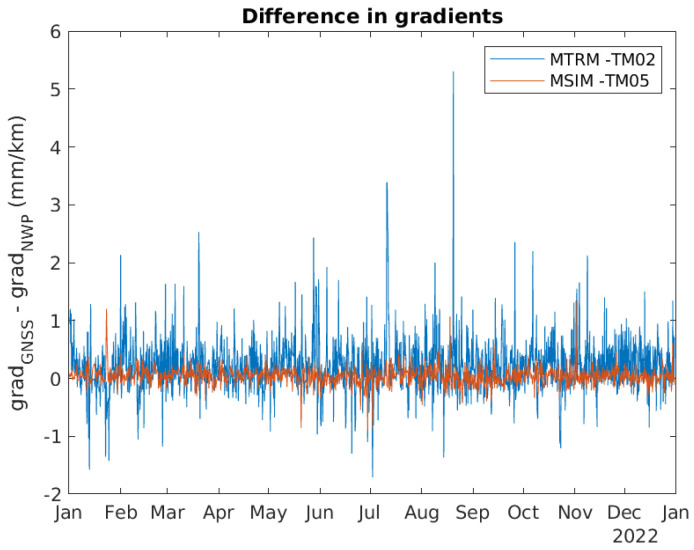
Difference between horizontal tropospheric gradients based on GNSS data and based on NWP data.

**Figure 15:**
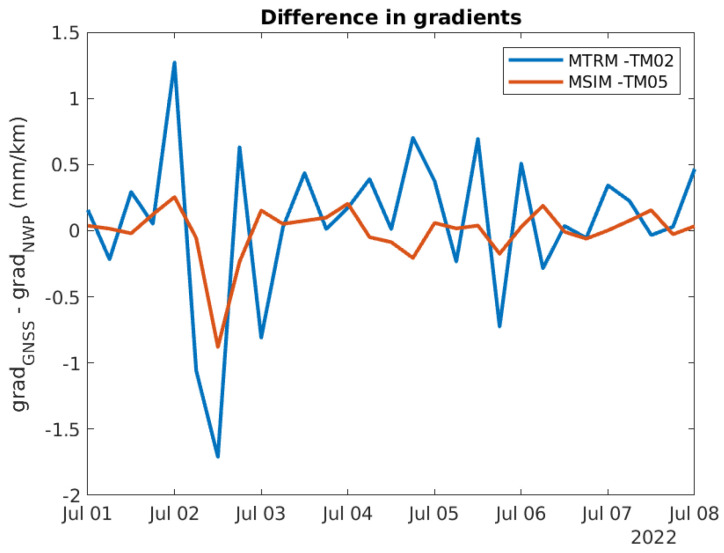
Zoomed in version of Figure 14.

Figure 14 contains the difference between the two gradients based on GNSS and NWP data. The difference seems to be the smallest for the MSIM-TM05 gradient, which has a long baseline. Figure 15 contains a zoomed-in version of Figure 14.

The mean and standard deviation of the difference in gradients are included in Table 14 and Table 15. The numbers for the two gradients MTRM-TM02 and MSIM-TM05 confirm what is observed from the curves, i.e., that the difference is smaller for the long baselines such as MSIM-TM05.

The correlation between the GNSS-based and NWP-based gradient estimates is shown in Table 16. For all gradients, the correlation is weak or very weak. The largest correlation coefficient is for the largest baseline.

#### 3.6.2. Differential ZWD between Reference Stations

To investigate the noise level in estimating gradients using NWP data, it can be convenient to instead evaluate the differential ZWD. Table 17 and Table 18 contain the mean values of the dZWD based on GNSS and NWP data, respectively. We see that the dZWD for reference stations closely located like MTRM, TM02 and TM03 is quite small compared to the dZWD for stations far apart such as MSIM and TM05, and also that the dZWD is relatively large for reference stations at different heights such as MTRM and TM05.

**Figure 16:**
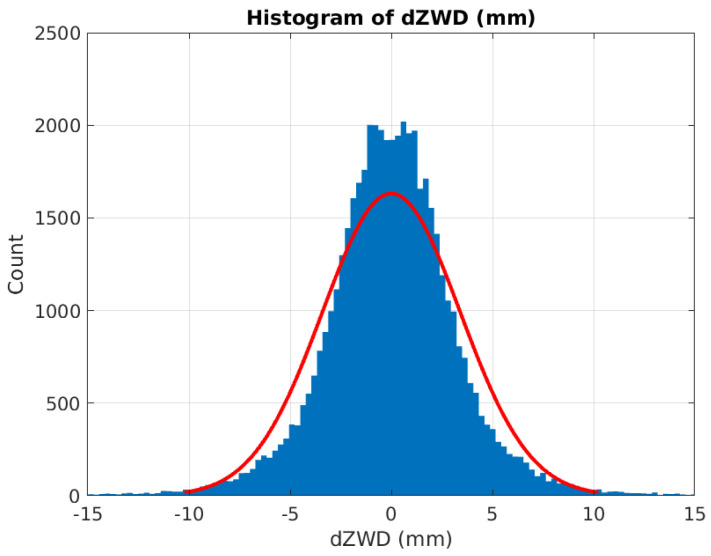
Histogram over dZWD between pairs of reference stations. ZWD estimates are based on NWP data.

The histogram of the dZWD timeseries for all stations is shown in Figure 16. These timeseries are calculated using NWP data. For most of the samples, the difference is below 5 mm, and for almost all, it is below 10 mm. This can be compared to the difference between estimates of the ZWD based on GNSS and NWP data (see Figure 11), which is much more variable, commonly up to 60 mm with occasional excursions to 80 mm. If we assume the ZWD data based on GNSS are ground truth (or at least much more accurate than those based on NWP data), this means that the error in the ZWD data based on NWP data typically is much higher than the difference in ZWD for the different stations. This may be the reason why much of the information regarding true gradients is lost when using NWP data.

#### 3.6.3. Detection of Largest Gradients

**Figure 17:**
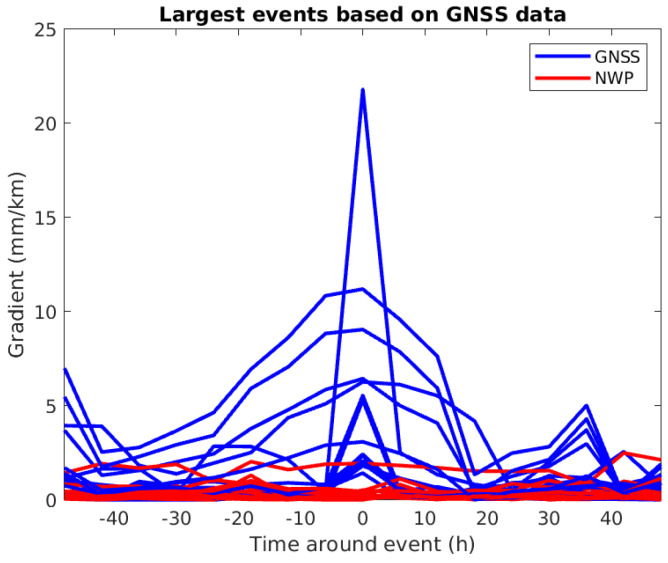
Timeseries around the largest events based on GNSS data (see Table 19).

The results so far indicate that gradient estimation based on NWP is unreliable because of the high noise level in the NWP data. However, we are mainly interested in the detection of the largest gradients. For most of the data, the gradients are low, and therefore, buried in noise. But if we can detect the steepest gradients, using NWP data may still be useful. Table 19 lists the largest estimates based on GNSS data for each of the 15 gradients together with the corresponding estimates based on NWP data. There is little indication that the events detected using GNSS data can also be detected using NWP data. This is confirmed by inspecting the timeseries surrounding the events (see Figure 17). In the figure, 0 on the x-axis is the time of the largest gradient and the blue curves correspond to the largest events. The red curves corresponding to the gradients based on NWP data do not show any indication of detecting the events. The same exercise is conducted including more than one event per station, with the same result.

**Figure 18:**
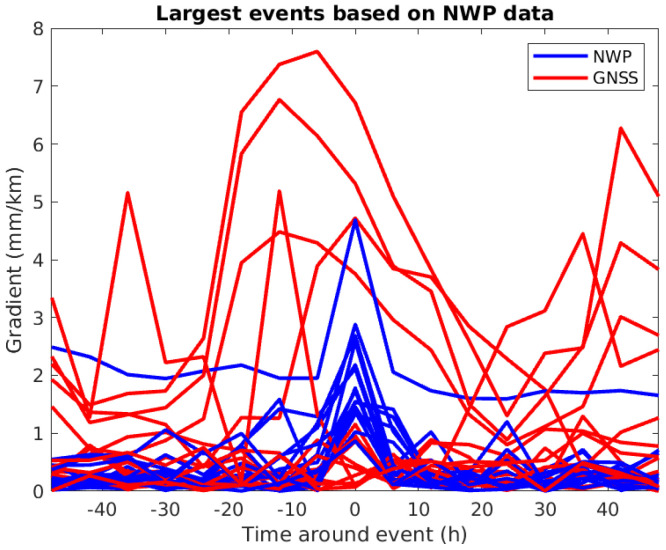
Timeseries around the largest events based on NWP data (see Table 20).

Table 20 shows the inverse situation. The largest events based on NWP data are listed with the corresponding estimates based on GNSS data. We see that in this case, the estimates based on GNSS data are also relatively high. Figure 18 shows the timeseries around these events, and it seems as though the level of the gradients based on GNSS data, in general, is higher and that it is difficult to corroborate that events identified based on NWP data also can be detected using GNSS data.

### 3.7. Gradients Calculated at Each Station

The data from the GipsyX software include the northern and eastern components of the gradients calculated at each of the stations. It can be of interest to compare the magnitude of the gradients estimated by each station with the gradients calculated from the difference between ZWD values at the two stations. One hypothesis is that the gradient at a position may be higher than the first-order approximation of the gradient over the baseline between two stations.

Table 21 shows the mean values of the two components and the total gradient. The mean of the total gradient varies between 0.55 mm/km and 0.65 mm/km. The values are generally higher than those calculated from the difference in ZWD values (see Table 12 and Table 13), although a few of those gradients are higher. Table 22 shows the maximum values of the gradients, and they range from 2.44 mm/km to 3.16 mm/km. This is low compared to some of the maximum gradients found from GNSS-based ZWD values (see Table 20) but in the same order as maximum gradients found from NWP-based ZWD values (see Table 19).

A more thorough comparison between the gradients estimated at each reference station and gradients between reference stations can be conducted, for instance, concentrating on one reference station. The gradients towards each of the other reference stations could be compared to the gradient estimated at the reference station, and even splitting all gradients into eastern and northern components. The correlation between the two approaches could then be compared. Events with high gradients can also be considered more closely. None of this has currently been conducted but may be considered in a continuation of the work.

## 4. Considering ZWD and Gradients during the Storm Hans

In this section, we look into the estimated small-scale tropospheric delays and gradients during the storm Hans in August 2023. This was an extreme event by Norwegian standards when it came to rainfall. The purpose is to see if this also can be classified as an extreme event when considering tropospheric delays and gradients.

### 4.1. Information about the Storm Hans and NWP Data

Hans hit the southeastern parts of Norway during the days 7–9 August 2023 and caused floods in large parts of the region, and also further to the south along the rivers floating towards the Oslo fjord. Vang in Valdres was the station with the highest registered rainfall. From Norway MET’s website http://www.yr.no (accessed on 7 August 2024) we can see that it measured 88.5 mm and 43.8 mm of rain on the 8 and 9 of August, respectively. Unfortunately, measured rain per hour is not available for these dates.

### 4.2. The NWP Data

**Figure 19:**
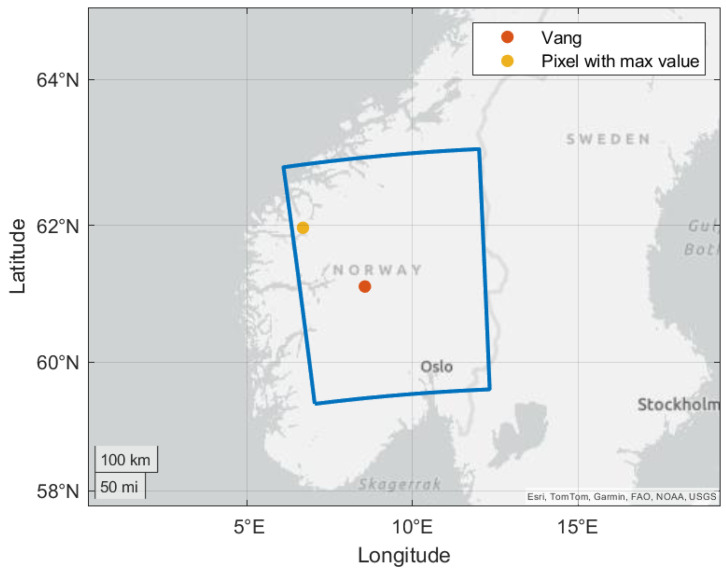
Map showing the area mostly affected by the storm. The blue bounding box represents the area considered for the NWP data.

We consider the area marked by the blue bounding box in Figure 19, as it covers the area with the highest measured rainfall. The number of pixels is 121×156 and the area covers about 240×320 km.

### 4.3. ZWD Estimates Based on NWP Data

**Figure 20:**
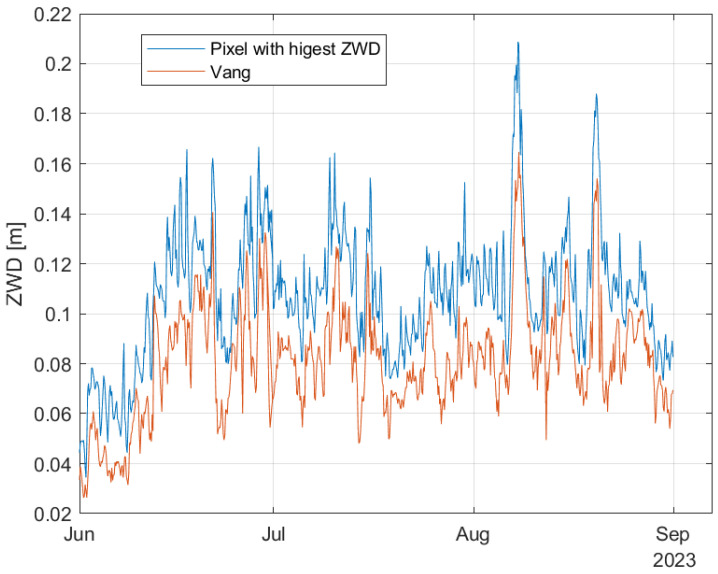
Estimated ZWD at Vang and the pixel with highest ZWD during the summer months of 2023.

**Figure 21:**
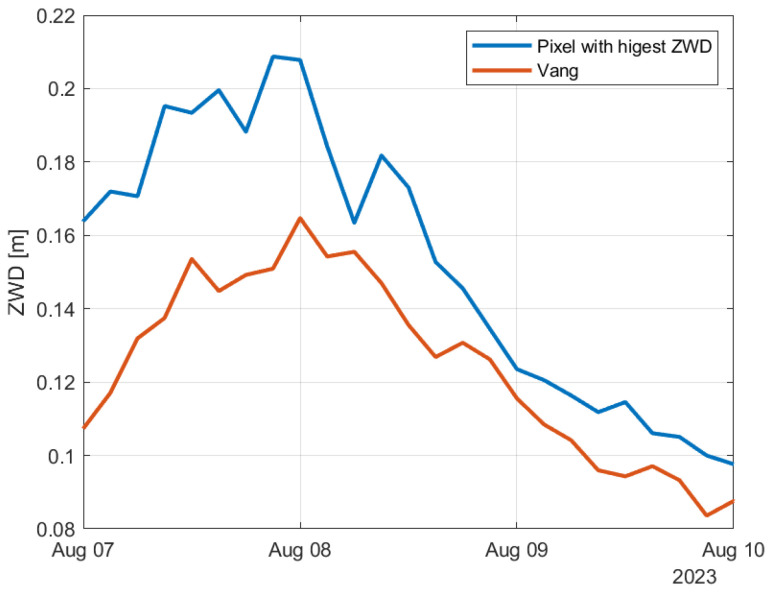
Estimated ZWD at Vang and the pixel with highest ZWD during Hans.

Now we look at the ZWD data, which are estimated from temperature, pressure and relative humidity. Figure 20 shows the ZWD for the pixel with the highest ZWD at each time sample and for Vang. Both curves have maximum values during Hans. Figure 21 shows the zoomed-in curves for the time period of the storm. The curves have maximum values late in the day the 7 and early the 8 of August local time. The curve for Vang is quite close to the curve corresponding to the maximum ZWD values at each time sample. The second peak we can see in the figure occurs on the 20th of August when Vang received 21 mm of rain.

### 4.4. Tropospheric Gradient Based on NWP Data

The tropospheric gradient is calculated for each of the pixels within the area by comparing the difference in ZWD to all the neighbouring pixels and then dividing by the baseline distance.

**Figure 22:**
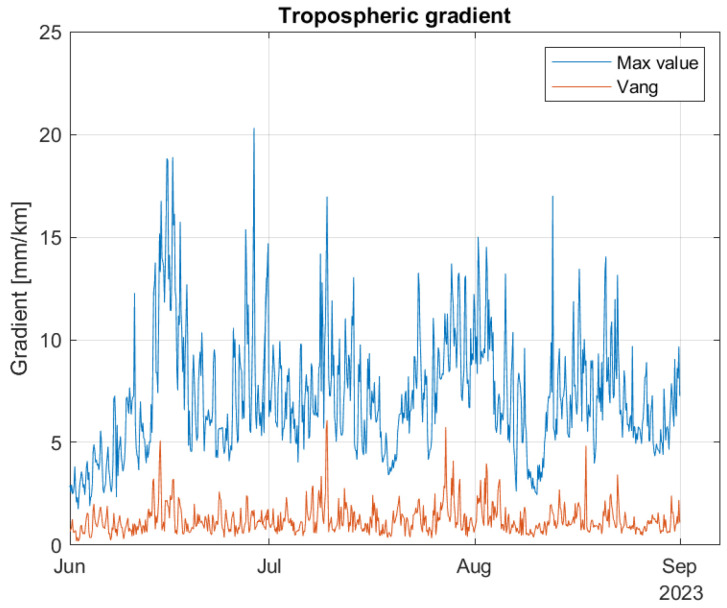
Tropospheric gradient for the summer months, including the maximum value within the coverage area and the pixel closest to Vang.

**Figure 23:**
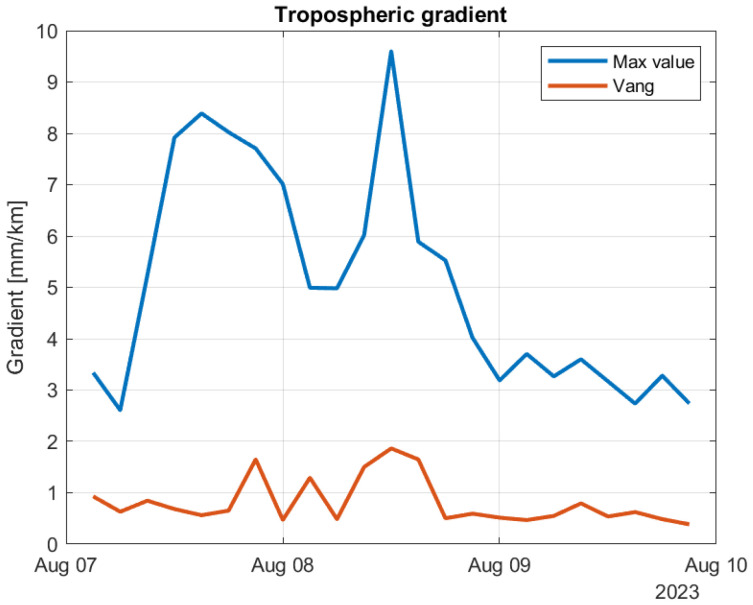
Same as the previous figure zoomed in to the time period of Hans.

Figure 22 contains two curves. The blue curve contains the maximum value within the coverage area for each of the time samples. The red curve corresponds the gradient value at Vang. We see that the maximum estimated gradient is 20.3 mm/km and occurred on June 28. Considering Vang, the highest estimated gradient is 6.1 mm/km and occurred July 9. In Figure 23, the time period of Hans is considered. The maximum value is 9.6 mm/km and occurred at 12 h on 8 August. For Vang, the maximum gradient is 1.8 mm/km, also occurring at 12 h on 8 August. Based on these curves, the storm Hans did not lead to any noticeable increase in tropospheric gradients. This is in contrast to the ZWD values, that did increase and reached the highest values for the year by a good margin for the affected areas.

## 5. Concluding Remarks

This paper addresses three questions:

- What is the largest gradient of zenith wet delay in the horizontal direction we can detect on short baselines within the considered area based on NWP data?
- Can we trust the gradient estimates based on NWP data?
- Is there an observable increase in the gradient of zenith wet delay in the horizontal direction during a strong storm with heavy rainfall leading to sustained flooding?

Concerning the first question, horizontal gradients up to 50 mm/km have been detected within the considered area based on NWP data. This is based on the analysis of three consecutive years of data from 2021 to 2023. The temporal resolution of the NWP data is in the order of hours, which is much lower than what can, e.g., be obtained from GNSS reference stations. It is likely that higher gradients would be detected with a higher sampling rate.

To address the second question, gradients estimated using NWP data are compared to gradients estimated using the GipsyX software and GNSS measurements for a cluster of reference stations located in the Tromsø area. Although the correlation between the ZWD estimates using the two data sources is strong, the correlation in estimated gradients is weak. The most probable cause of this is that the NWP data are not sufficiently accurate. The correlation between ZWD data at the different reference stations is very strong with a correlation coefficient in the order of 0.990–0.999. The correlation between GNSS-based and NWP-based estimates for the same gradients is not that strong with correlation coefficients around 0.82. The ZWD estimates based on NWP data may then be so noisy that it is not possible to detect the small differences in ZWD causing the gradients. Even the steepest gradients detected based on GNSS data seem difficult to detect using NWP data. Another factor that may have an impact is that the GNSS-based estimates are far lower noise but contain at least one artifact which complicates the comparison. Some ZWD estimates based on GNSS data are negative due to processing artifacts, which may also lower the apparent correlation between the two data sources even while the GNSS data remain highly self-consistent.

Concerning the last question, the estimated ZWD did increase and reach a peak during the considered storm event. However, no notable increase in the horizontal tropospheric gradient values was observed during the storm based on the NWP data. The reason for this may be that there was no increase. If there was an increase it is, however, likely that it was not detected due to inaccuracies in the NWP data as explained in the previous paragraph.

## Figures and Tables

**Table 1:** Location of reference stations.

Station	Latitude [deg]	Longitude [deg]
MSIM	69.8355	19.0106
MTRM	69.6627	18.9396
TM02	69.6947	18.9828
TM03	69.7049	19.0014
TM04	69.7431	19.1366
TM05	69.5756	18.7846

**Table 2:** Distance between the reference stations.

Station	MSIM	MTRM	TM02	TM03	TM04	TM05
MSIM	0.0	19.5	15.7	14.6	11.4	30.3
MTRM		0.0	3.9	5.3	11.8	11.4
TM02			0.0	1.3	8.0	15.4
TM03				0.0	6.7	16.7
TM04					0.0	23.1
TM05						0.0

**Table 3:** Distance between closest grid points in km.

Station	MSIM	MTRM	TM02	TM03	TM04	TM05
MSIM	0.0	17.6	14.9	14.9	11.1	30.8
MTRM		0.0	3.5	3.5	10.6	13.4
TM02			0.0	0.0	7.0	16.7
TM03				0.0	7.0	16.7
TM04					0.0	23.5
TM05						0.0

**Table 4:** Distance from the reference stations to the closest grid point as well as the heights of the reference stations and the corresponding closest grid points in m.

Station	Dist (m)	Height Stations (m)	Height Surface (m)
MSIM	778	62.8	74.6
MTRM	1437	138.1	3.9
TM02	285	131.8	16.3
TM03	1194	180.3	16.3
TM04	809	51.5	57.9
TM05	1219	39.3	18.3

**Table 5:** Mean ZWD value for the stations in mm.

Station	GNSS	NWP Surface	NWP Station Height	NWP Interpolated
MSIM	68.1	72.5	72.5	71.5
MTRM	65.9	75.8	71.9	71.8
TM02	66.6	75.5	72.2	72.2
TM03	64.8	75.5	70.8	70.4
TM04	70.9	73.7	73.7	73.5
TM05	72.3	74.9	74.6	73.9
All	68.1	74.6	72.6	72.2

**Table 6:** Mean difference between ZWD based on GNSS and NWP data in mm.

Station	NWP Surface	NWP Station Height	NWP Interpolated
MSIM	−4.5	−4.5	−3.5
MTRM	−10.0	−6.1	−6.0
TM02	−9.0	−5.7	−5.6
TM03	−10.8	−6.1	−5.7
TM04	−2.9	−2.9	−2.7
TM05	−2.6	−2.4	−1.7
All	−6.6	−4.6	−4.2

**Table 7:** Standard deviation of the difference between ZWD based on GNSS and NWP data in mm.

Station	NWP Surface	NWP Station Height	NWP Interpolated
MSIM	28.9	28.9	29.0
MTRM	28.2	28.2	28.2
TM02	28.3	28.3	28.3
TM03	28.2	28.2	28.2
TM04	28.9	28.9	28.9
TM05	28.6	28.6	28.6
All	28.7	28.6	28.6

**Table 8:** Interpretation of Pearson correlation coefficient values.

ρ Interval	Relationship Level
0.80–1	Very strong
0.60–0.799	Strong
0.40–0.399	Moderate
0.20–0.399	Weak
0–0.199	Very week

**Table 9:** Correlation between ZWD estimates at the different reference stations based on NWP data.

	MSIM	MTRM	TM02	TM03	TM04	TM05
MSIM	1.00000	0.99642	0.99693	0.99721	0.99782	0.99411
MTRM	0.99642	1.00000	0.99956	0.99939	0.99844	0.99811
TM02	0.99693	0.99956	1.00000	0.99989	0.99900	0.99763
TM03	0.99721	0.99939	0.99989	1.00000	0.99918	0.99753
TM04	0.99782	0.99844	0.99900	0.99918	1.00000	0.99659
TM05	0.99411	0.99811	0.99763	0.99753	0.99659	1.00000

**Table 10:** Correlation between ZWD estimates at the different reference stations based on GNSS data.

	MSIM	MTRM	TM02	TM03	TM04	TM05
MSIM	1.00000	0.99703	0.99721	0.99684	0.99046	0.99548
MTRM	0.99703	1.00000	0.99897	0.99851	0.99149	0.99807
TM02	0.99721	0.99897	1.00000	0.99898	0.99166	0.99798
TM03	0.99684	0.99851	0.99898	1.00000	0.99158	0.99760
TM04	0.99046	0.99149	0.99166	0.99158	1.00000	0.99075
TM05	0.99548	0.99807	0.99798	0.99760	0.99075	1.00000

**Table 11:** Correlation coefficient between NWP based and GNSS-based ZWD estimates.

Reference Station	Correlation Coefficient
MSIM	0.81810
MTRM	0.82352
TM02	0.82316
TM03	0.82336
TM04	0.81850
TM05	0.82783

**Table 12:** Mean value of gradient based on GNSS data between two stations (mm/km).

Station	MSIM	MTRM	TM02	TM03	TM04	TM05
MSIM	0.00	0.18	0.19	0.29	0.38	0.16
MTRM		0.00	0.44	0.41	0.49	0.57
TM02			0.00	1.69	0.63	0.38
TM03				0.00	0.99	0.46
TM04					0.00	0.17
TM05						0.00

**Table 13:** Mean value of gradient based on NWP data between two stations (mm/km).

Station	MSIM	MTRM	TM02	TM03	TM04	TM05
MSIM	0.00	0.13	0.15	0.16	0.24	0.13
MTRM		0.00	0.23	0.31	0.19	0.23
TM02			0.00	1.34	0.22	0.17
TM03				0.00	0.48	0.23
TM04					0.00	0.11
TM05						0.00

**Table 14:** Mean value of difference between gradients based on NWP and GNSS data (mm/km).

Station	MSIM	MTRM	TM02	TM03	TM04	TM05
MSIM	0.00	0.05	0.04	0.12	0.14	0.04
MTRM		0.00	0.21	0.10	0.30	0.34
TM02			0.00	0.35	0.40	0.21
TM03				0.00	0.51	0.22
TM04					0.00	0.06
TM05						0.00

**Table 15:** Standard deviation of difference between gradients based on NWP and GNSS data (mm/km).

Station	MSIM	MTRM	TM02	TM03	TM04	TM05
MSIM	0.00	0.19	0.22	0.25	0.56	0.15
MTRM		0.00	0.47	0.46	0.51	0.31
TM02			0.00	1.52	0.74	0.23
TM03				0.00	0.89	0.23
TM04					0.00	0.27
TM05						0.00

**Table 16:** Correlation between the GNSS-based and NWP-based gradient estimates.

Id	Gadient	$\rho_{\mathrm{GNSS},\mathrm{NWP}}$
1	MSIM-MTRM	0.12
2	MSIM-TM02	0.09
3	MSIM-TM03	0.12
4	MSIM-TM04	0.10
5	MSIM-TM05	0.34
6	MTRM-TM02	0.03
7	MTRM-TM03	0.04
8	MTRM-TM04	0.13
9	MTRM-TM05	0.22
10	TM02-TM03	0.05
11	TM02-TM04	0.14
12	TM02-TM05	0.24
13	TM03-TM04	0.15
14	TM03-TM05	0.28
15	TM04-TM05	0.11

**Table 17:** Mean value of dZWD based on GNSS data between two stations (mm).

Station	MSIM	MTRM	TM02	TM03	TM04	TM05
MSIM	0.00	3.46	3.02	4.16	4.34	4.96
MTRM		0.00	1.74	2.15	5.77	6.48
TM02			0.00	2.28	5.04	5.79
TM03				0.00	6.69	7.61
TM04					0.00	4.01
TM05						0.00

**Table 18:** Mean value of dZWD based on NWP data between two stations (mm).

Station	MSIM	MTRM	TM02	TM03	TM04	TM05
MSIM	0.00	2.49	2.37	2.38	2.68	3.80
MTRM		0.00	0.90	1.62	2.28	2.65
TM02			0.00	1.81	1.78	2.58
TM03				0.00	3.22	3.86
TM04					0.00	2.56
TM05						0.00

**Table 19:** Listing the maximum estimated value for each gradient using GNSS data, and the corresponding estimated values using NWP data.

Gradient	Timestamp	Grad Based on GNSS (mm/km)	Grad Based on NWP (mm/km)
MSIM-MTRM	23-January-2022 12 h	1.80	0.01
MSIM-TM02	1-November-2022 18 h	2.43	0.08
MSIM-TM03	1-November-2022 18 h	2.42	0.07
MSIM-TM04	29-October-2022 12 h	6.27	0.27
MSIM-TM05	1-November-2022 18 h	1.43	0.09
MTRM-TM02	20-August-2022 0 h	5.55	0.24
MTRM-TM03	10-October-2022 0 h	5.31	0.53
MTRM-TM04	29-October-2022 18 h	6.44	0.02
MTRM-TM05	18-August-2022 0 h	2.33	0.20
TM02-TM03	10-October-2022 0 h	21.79	1.94
TM02-TM04	29-October-2022 18 h	9.05	0.23
TM02-TM05	20-August-2022 0 h	1.93	0.24
TM03-TM04	29-October-2022 18 h	11.20	0.48
TM03-TM05	20-August-2022 0 h	2.07	0.44
TM04-TM05	29-October-2022 18 h	3.09	0.02

**Table 20:** Listing the maximum estimated value for each gradient using NWP data, and the corresponding estimated values using GNSS data.

Gradient	Timestamp	Grad Based on NWP (mm/km)	Grad Based on GNSS (mm/km)
MSIM-MTRM	25-June-2022 0 h	1.33	0.96
MSIM-TM02	25-June-2022 0 h	1.55	1.16
MSIM-TM03	22-October-2022 18 h	1.46	0.27
MSIM-TM04	22-October-2022 18 h	1.73	4.72
MSIM-TM05	25-June-2022 0 h	1.02	0.86
MTRM-TM02	23-October-2022 12 h	2.12	0.91
MTRM-TM03	23-October-2022 12 h	2.59	0.09
MTRM-TM04	23-October-2022 6 h	1.53	3.75
MTRM-TM05	29-June-2022 12 h	2.18	0.46
TM02-TM03	28-July-2022 12 h	4.68	0.09
TM02-TM04	23-October-2022 6 h	2.88	5.32
TM02-TM05	23-October-2022 6 h	1.78	0.07
TM03-TM04	23-October-2022 6 h	2.69	6.71
TM03-TM05	29-June-2022 12 h	1.37	0.40
TM04-TM05	02-July-2022 12 h	0.87	0.14

**Table 21:** Mean gradients at each station, including Eastern and Northern components.

Station	Mean(E) (mm/km)	Mean(N) (mm/km)	Mean(Grad) (mm/km)
MSIM	−0.13	−0.21	0.65
MTRM	−0.06	−0.29	0.59
TM02	−0.22	−0.22	0.58
TM03	−0.19	−0.23	0.59
TM04	−0.14	−0.14	0.55
TM05	−0.13	−0.26	0.60

**Table 22:** Max gradients in mm/km at each station, including Eastern and Northern components.

Station	Max(E) (mm/km)	Max(N) (mm/km)	Max(Grad) (mm/km)
MSIM	2.63	2.98	3.16
MTRM	2.13	2.57	2.60
TM02	2.46	2.24	2.55
TM03	2.00	2.34	2.52
TM04	2.19	2.36	2.44
TM05	2.84	2.98	3.07

## Data Availability

Data are contained within the article.
